# Metabolic Response of Peach Fruit to Invasive Brown Marmorated Stink Bug (*Halyomorpha halys* Stål.)’s Infestation

**DOI:** 10.3390/ijms25010606

**Published:** 2024-01-03

**Authors:** Sasa Gacnik, Denis Rusjan, Maja Mikulic-Petkovsek

**Affiliations:** Department of Agronomy, Biotechnical Faculty, University of Ljubljana, Jamnikarjeva 101, SI-1000 Ljubljana, Slovenia; sasa.gacnik@bf.uni-lj.si (S.G.); denis.rusjan@bf.uni-lj.si (D.R.)

**Keywords:** *Prunus persica* (L.) Batsch, Pentatomidae, flavonoids, phenylpropanoid pathway, plant defence, induced resistance

## Abstract

The brown marmorated stink bug (BMSB; *Halyomorpha halys* Stål.) is a highly destructive and polyphagous invasive pest that poses a serious threat to more than a hundred reported host plants. In the current study, the metabolic response of peach fruit of two cultivars—‘Maria Marta’ and ‘Redhaven’—to BMSB infestation was studied using high-performance liquid chromatography (HPLC) and mass spectrometry (MS). In general, a strong phenolic response to BMSB infestation in peach flesh in the injury zone was observed, with flavanol content increasing by 2.4-fold, hydroxycinnamic acid content by 5.0-fold, flavonol content by 3.2-fold, flavanone content by 11.3-fold, and dihydrochalcones content by 3.2-fold compared with the undamaged tissue in the cultivar ‘Maria Marta’. The phenolic response in the ‘Redhaven’ cultivar was even stronger. Consequently, the total phenolic content in the injured flesh also increased, 3.3-fold in ‘Maria Marta’ and 6.9-fold in ‘Redhaven’, compared with the uninjured flesh. Infestation with BMSB induced the synthesis of cyanidin-3-glucoside, which is not normally present in peach flesh. In comparison, the phenolic response was lower in peach peel, especially in the cultivar ‘Maria Marta’. The study showed that both peach cultivars reacted to BMSB infestation with an increase in phenolic content in the peach flesh, but in a limited area of injury.

## 1. Introduction

The brown marmorated stink bug (BMSB; *Halyomorpha halys* Stål., 1855; Hemiptera: Pentatomidae) is a highly invasive pest that poses a serious global threat, especially to regions with intensive fruit and vegetable production [1]. More than 100 host plants are known, with a preference for plants from the Rosaceae and Fabaceae families [2].

BMSB is native to Japan, Korea, China, and Taiwan [3]. It was introduced and spread in North America in the mid-1990s [4] and in Europe in 2004 [5]. BMSB was first reported in Slovenia in the spring of 2017 [6]. Subsequent years quickly followed reports on the effect of its infestation on the reduced physical and chemical properties of fruit species and their metabolic response to BMSB [7,8].

**Figure 1:**
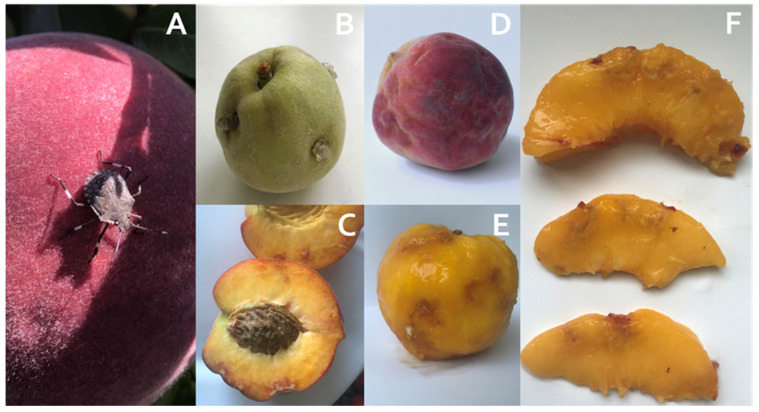
(**A**) Late nymphal stage of a brown marmorated stink bug (BMSB), showing banding on legs and antennae. (**B**) Gummnosis on a young peach. (**C**) Internal damage in a peach. (**D**) External damage in a peach. (**E**) Damaged peach under the peel. (**F**) Depth of internal damage by BMSB in peach.

BMSB adults are shield-shaped and brown-mottled, with two light bands on dark antennae and a typical band pattern on the margins of the abdomen (connexivum) [4,6]. Unlike native stink bug pests, damage can be caused by both adult BMSB and nymphs (Figure 1A) by piercing the surface, injecting digestive enzymes, and sucking up plant fluids, usually resulting in suberification, the formation of necrotic areas, and the deliquescence of the fruit flesh [2,9,10]. Gummosis—gummy, whitish exudate—may be present on stone fruits [11]. Fruits damaged by BMSB are not suitable for sale, not only because of their appearance but also because of the unpleasant odour caused by the contamination with *trans*-2-octenal and *trans*-2-decenal secreted by BMSB when threatened [12,13].

On peaches, BMSB can complete the full development cycle, which is why they are amongst their most preferred host plants [9,14,15]. Most damage is caused to fruits, which varies depending on the life stage of BMSB and the time of infestation [11]. Some typical external and internal injuries on peach fruit caused by BMSB are shown in Figure 1B–F. According to Damos et al. (2020) [15], the percentage of peach damage caused by BMSB in 2018 and 2019 was 63 ± 3% and 25 ± 5%, respectively, mainly in the form of fruit deformation, which in most cases resulted in suberification. Acebes-Doria et al. (2016) [11] compared feeding injuries on harvested peaches caused by young and older nymphs and adults present in early June and late July. They concluded that the highest percentage of injured fruits and injuries per peach fruit in the early season was caused by adult feeding, with more internal than external injuries and gummosis occurring only in this combination. Joseph et al. (2015) [16] also reported a greater extent of internal injuries.

Plants are capable of developing or activating defence mechanisms against a new invasive species that respond similarly to native herbivore species, or they can recognise the invader with a novel response [17,18,19], usually triggered by hatching a herbivore’s eggs on the plant [18,20], with elicitors present in the eggs or the exocrine secretions and covering the eggs [21]. In general, defences that allow plants to control a herbivore attack can be physical or chemical barriers (semiochemicals and secondary metabolites like plant toxins or plant nutrients), described in more detail by Peterson et al. (2016) and War et al. (2012) [19,22].

Plant secondary metabolites, mainly phenolic compounds [23,24], play an important role in the development of direct plant defences [22]. They can be in the form of phytoanticipins, which are constantly present in plants and whose content usually increases sharply when plants are attacked by pests, or phytoalexins, which are sensitised from precursors in response to a pest attack, or inactive bound forms, which are readily converted to a biologically active free compound upon a pest attack and are much more toxic [19,23].

Knowledge of the metabolic response of peaches damaged by BMSB is insufficient, which this study attempted to address. Two tissues of undamaged and damaged peach fruits were studied (peel and flesh), and two cultivars—‘Maria Marta’ and ‘Redhaven’—were included for better representativeness. The aim of the study was, therefore, to determine how BMSB affects the metabolic response at the level of phenolic compounds in peach fruit peel and flesh, as well as primary metabolites in the flesh. It was also of interest to determine whether BMSB elicited a limited response only at the damaged site or whether it was also present in the seemingly undamaged part of the fruit.

## 2. Results

### 2.1. Visual Appearance of Peaches Damaged by Brown Marmorated Stink Bug (BMSB)

Injuries caused by BMSB appeared as surface depressions with a distorted, dented, and discoloured appearance and red flesh under the surface (Figure 1C–F). The size of surface depressions on the peach fruit with peel (Figure 1D) ranged in diameter from 0.8 to 9.5 cm, with many depression zones converging. After the removal of the peel (Figure 1E), individual zones were visible, ranging in size from 0.5 to 6 cm. The depth of the damages can be seen in Figure 1F—they ranged from 0.5 cm to 1.8 cm.

**Figure 2:**
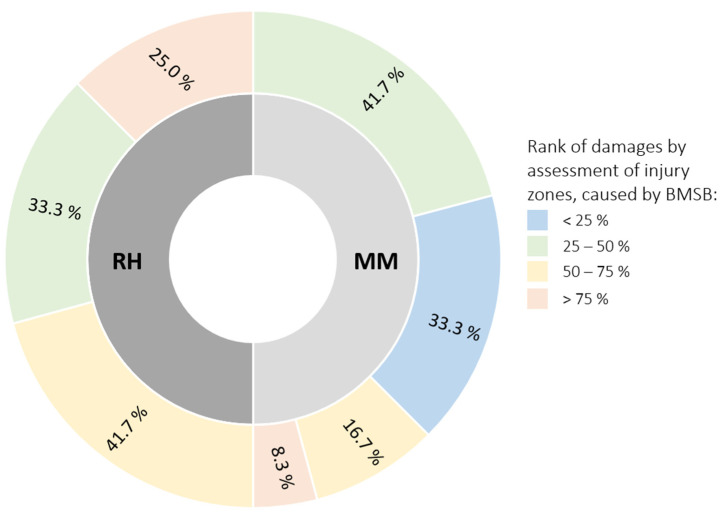
Rank of damages (% of fruits in one rank), based on surface depressions caused by a brown marmorated stink bug (BMSB) on two peach cultivars—‘Maria Marta’ (MM) and ‘Redhaven’ (RH).

For the cultivar ‘Maria Marta’, the majority (75%) of the fruits were in the <25% and 25–50% whole fruit surface depression injury classes, with 25% of fruits in the >50% injury zone (Figure 2). While in ‘Redhaven’, more than half (66.7%) of the fruits were classified in the damage class of surface impression of more than 50% of the fruit surface. In this research, the damage was to a great extent caused on the sun-exposed part of the peach fruits in both cultivars.

### 2.2. Effects of BMSB Infestation on Sugar and Organic Acid Content in Peach Fruit

The contents of sugars and organic acids in peaches damaged and undamaged by BMSB are shown in Table 1. In general, BMSB infestation did not exert excessive effects on sugar and organic acid content. The exception was the reduced content of sucrose, sorbitol, and, consequently, total sugars analysed in the damaged tissue of the cultivar ‘Maria Marta’ and the increased content of quinic and shikimic acids in the damaged tissue of the cultivar ‘Redhaven’. The mean values of quinic and shikimic acids in ‘Maria Marta’ in the injured tissue increased, but ANOVA showed no statistically significant differences (*p* > 0.05). Sorbitol content was reduced by 52% in the injured tissue of ‘Maria Marta’ compared with the control and by 41.2% in the intact tissue of the injured peaches. Similarly, sucrose was reduced by up to 46.9% in the injured tissue compared with the intact tissue and by 39.9% compared with the control. BMSB consequently also affected the loss of total sugars, which was 36.7% lower compared with the control. In the ‘Redhaven’ cultivar, the injured tissue of the damaged peaches had a 3.7-fold higher content of quinic acid than the control tissue and a 2.0-fold higher content compared with the intact tissue of the damaged fruits. The content of shikimic acid in the injured tissue of the fruits damaged by BMSB was 4.1-fold higher compared with the undamaged tissue (both the control and the intact tissue).

### 2.3. Effects of BMSB Infestation on Phenolic Content in Peach Pulp

Forty-eight individual phenolic compounds (Table 2), belonging to 6 different phenolic groups—16 representatives of hydroxycinnamic acids (HCA), 9 flavanols (FLA), 17 flavonols (FLO), 3 flavanones (FLN), 1 dihydrochalcone (DHC), and 1 anthocyanin (ANT)—were identified in the peach flesh of both cultivars (Table 2). Amygdalin was also quantified in traces with HPLC-MS analysis, belonging to cyanogenic glycosides.

**Figure 3:**
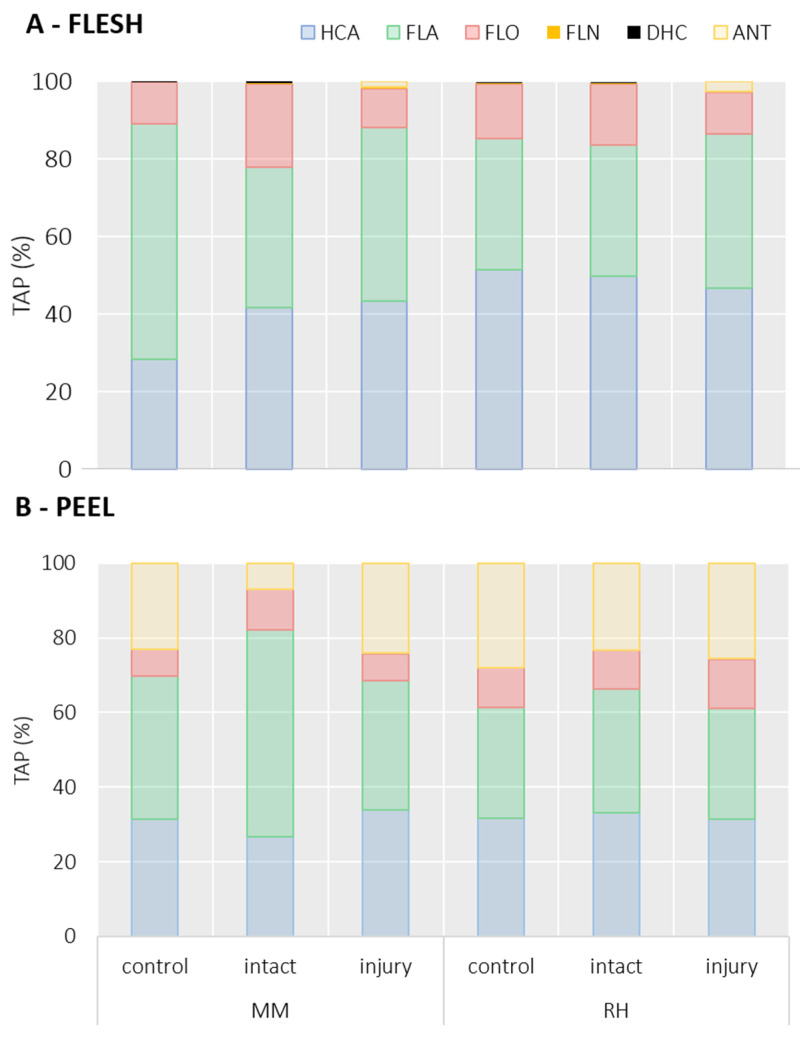
The content of phenolic groups (HCA: hydroxycinnamic acids, FLA: flavanols, FLO: flavonols, FLN: flavanones, DHC: dihydrochalcones, ANT: anthocyanins) according to total phenolic content (TAP) proportions (%) in the peach flesh (**A**) and peel (**B**) in different tissues, according to the injuries caused by a brown marmorated stink bug—control (tissue from undamaged peaches), intact (undamaged tissue from damaged peaches), and injured (tissue of damaged peaches from the injury zone in the surface depression). Two peach cultivars are presented—‘Maria Marta’ (MM) and ‘Redhaven’ (RH).

In the peach flesh of the cultivar ‘Maria Marta’, the largest share of TAP was represented by FLA with 60.9% TAP in the control tissue (Figure 3A) with a content of 97.85 ± 15.42 mg/kg FW (Table 1). In the peaches damaged by BMSB, the share of FLA decreased to 36.0% of TAP in the intact tissue and to 44.7% TAP in the injured tissue. The FLA content in the intact tissue of ‘Maria Marta’ was not different from that of the control, whereas the content in the injured tissue was 2.39 times higher compared with the control (234.3 ± 39.07 mg/kg FW; Table 1). The cultivar ‘Redhaven’ showed lower FLA content in the peach flesh (46.7 ± 12.03 mg/kg FW) and also lower FLA content in TAP (33.8%; Figure 3A) compared with the control cultivar ‘Maria Marta’. This did not alter the intact flesh of the damaged peach, while the FLA content in the injury zone increased significantly (*p* < 0.05) to 377 ± 35.44 mg/kg FW (8.1-fold higher than in the control). The total FLA share of TAP did not differ compared with the control of the ‘Redhaven’ cultivar. Procyanidin dimers were the most abundant FLA in the control peach pulp (Figure S1 and Table 1), followed by procyanidin trimers and catechin. The contents of all FLA significantly (*p* < 0.05) increased in the injured tissue, especially the content of catechin in both cultivars (MM: 31.42 ± 7.71; RH: 49.52 ± 6.45 mg/kg FW; Table 1), which was 9.9-fold higher in ‘Maria Marta’ and 10.2-fold higher in ‘Redhaven’ compared with the control tissue.

HCA were the most abundant phenolic group in the flesh of the control peaches of the cultivar ‘Redhaven’ (51.4% TAP; Figure 3A) and the second most abundant in ‘Maria Marta’ (27.9% TAP; Figure 3A). In the damaged fruits of the cultivar ‘Maria Marta’, the share of HCA significantly increased (*p* < 0.05) in both tissue zones (intact: 42.7% TAP; injury: 43.1% TAP; Figure 3A), while the share of HCA in the damaged fruits of ‘Redhaven’ was not changed compared to the control. Similar to FLA, HCA content increased in both cultivars in the injured tissue (MM: 228.9 ± 43.52; RH: 444.6 ± 31.21 mg/kg FW; Table 2), compared with the control and the intact tissues of the damaged fruits. In ‘Maria Marta’, the content of HCA in the injured tissue was 5.0-fold higher, and in ‘Redhaven’, it was 6.3-fold higher in comparison to the control. The intact tissue of the damaged fruits of both cultivars did not differ in HCA content compared to the control. Chlorogenic acid, followed by neochlorogenic acid (Table 2), was the most abundant HCA in the peach pulp of both cultivars. Both acids (*p* < 0.05) increased considerably in the injured tissues in both cultivars, whereas the intact tissue of the damaged fruits did not differ from the control in content. The increase was particularly pronounced in the content of chlorogenic acid, which was 9.9-fold higher in the cultivar ‘Maria Marta’ and 10.2-fold higher in ‘Redhaven’ (Table 2) than in the control tissue. The percentage of chlorogenic acid in the TAP also increased prominently (*p* < 0.05) in the injured tissue of both cultivars (Figure S1) by 18.3% in ‘Maria Marta’ and 8.8% in ‘Redhaven’ compared with the control. In comparison to the control, the content of 3-feruloylquinic acid in the injured pulp also increased strongly in both cultivars (MM: 10.3-fold; RH: 10.4-fold). In ‘Maria Marta’, dicaffeoylquinic acid 1 and dicaffeoylquinic acid 2 increased significantly (more than 8-fold), and in ‘Redhaven’, caffeic acid hexoside 1, caffeic acid hexoside 2, 3-*p*-coumaroylquinic acid, 5-*p*-coumaroylquinic acid, and sinapoylhexoside increased significantly.

The content of FLO in the control tissue was similar in both cultivars (MM: 16.98 ± 3.45; RH: 19.24 ± 4.43 mg/kg FW; Table 2), and in the injured tissue of both cultivars, it significantly increased (*p* < 0.05), especially in ‘Redhaven’. On the contrary, the FLO share of TAP was notably (*p* < 0.05) lower in the injured tissue (MM: 10.6%; RH: 10.7%) compared with the intact tissue (MM: 20.6%; RH: 15.2%; Figure 3). Quercetin-3-galactoside was the most represented FLO representative, with 8.00 ± 2.08 mg/kg FW in the control tissue of ‘Maria Marta’ and 12.16 ± 2.92 mg/kg FW in the control ‘Redhaven’. Values in both cultivars (*p* < 0.05) increased considerably in the injured tissues (MM: 2.8-fold; RH: 4-fold compared with the control).

FLN and DHC represented a very low proportion of TAP (<1%) in the peach flesh. All contents of individual representatives of both groups in the injured tissue were significantly increased (*p* < 0.05; Table 2). Cyanidin-3-glucoside, belonging to ANT, was also identified in the injured tissue of the peach flesh of both cultivars, with values of 7.09 ± 2.25 mg/kg FW (MM) and 23.24 ± 12.38 mg/kg FW (RH).

TAP in the flesh of the control tissue of ‘Maria Marta’ was 160.6 ± 27.83 mg/kg FW and 137.6 ± 33.44 mg/kg FW (Table 2) in ‘Redhaven’. Similar to most individual phenolics, TAP also greatly increased in the injured tissue of the BMSB-damaged fruits, especially in ‘Redhaven’ (6.9-fold compared with the control). In ‘Maria Marta’, TAP values of the injured tissue increased 3.3-fold compared with the control tissue.

### 2.4. Effects of BMSB Infestation on Phenolic Content in Peach Peel

Forty-nine individual phenolic compounds were identified (Table 3) in the peach peel of both cultivars, most of which were also present in peach pulp in lower contents. The content of TAP in the control peach peel of the cultivar ‘Maria Marta’ was 736.6 ± 100.5 mg/kg FW and of the cultivar ‘Redhaven’ was 1916 ± 463.1 mg/kg FW (Table 3). The content of TAP in the peel of the damaged fruits of the cultivar ‘Maria Marta’ did not differ from that of the control in either the injured or intact tissue, whereas the content of TAP in the cultivar ‘Redhaven’ was 1.3-fold higher in the injured tissue than in the control peel (Table 3).

HCA and FLA (Figure 3B) were the most represented phenolic groups in peach peel and quite equal in terms of TAP share (HCA: MM: 26.7–33.8% TAP; RH: 31.5–33.1% TAP, FLA: MM: 34.8–55.5% TAP; RH: 29.6–33.1% TAP), which in general did not significantly (*p* > 0.05) differ with respect to different tissues. The exception was the intact tissue of the cultivar ‘Maria Marta’, where a lower share of HCA (26.7%) and a higher share of FLA (55.5%) compared with the control and the injured tissue (Figure 3B) were observed. Individual representatives of HCA in ‘Maria Marta’ (Table 3) were not altered with respect to the tissue type in the damaged fruits. The exception was neochlorogenic acid, which was significantly (*p* < 0.05) lower in the intact tissue, and 5-CQA2, which was higher in the intact tissue compared with the control. In contrast, individual HCAs responded in the injured tissue of the cultivar ‘Redhaven’, and greatly (*p* > 0.05) increased compared with the control tissue (Table 3). In addition, the content of individual representatives and total FLA in ‘Maria Marta’ did not differ between different tissues either, while FLA content in the cultivar ‘Redhaven’ in the injured tissue (*p* < 0.05) increased by 56.2% compared with the control (Table 3). Between the intact and the injured tissues in the damaged fruits, there was no significant difference observed.

In peach peel, FLOs in ‘Maria Marta’ were present in a lower share, from 7.0 to 10.9% of TAP and in a higher percentage in the intact tissue of BMSB-damaged fruits, and in ‘Redhaven’ from 10.4 to 13.1% TAP, where a higher percentage was observed in the injured tissue (Figure 3B). The content of mostly all individual representatives and total FLOs did not differ between different tissues in ‘Maria Marta’ peel either (FLO content—control: 48.61 ± 4.61 mg/kg FW; intact: 57.6 ± 3.37 mg/kg FW; injury: 46.25 ± 8.2 mg/kg FW), while ‘Redhaven’s injured peel had 2.1-fold higher content compared with the control and 2.2-fold higher content compared with the intact peel.

ANT presented 23.1% of TAP in the control peel of the cultivar ‘Maria Marta’ and 28.1% of TAP in the control peel of ‘Redhaven’ (Figure 3B). The injured tissue of both cultivars and the intact tissue of the damaged peaches of the cultivar ‘Redhaven’ did not differ in ANT content when compared to the control, while the intact tissue of ‘Maria Marta’ had 4.7-fold (*p* < 0.05) lower content compared with the control (147.14 ± 30.54 mg/kg FW; Table 3). ANT share of TAP also decreased substantially (*p* < 0.05) in the ‘Maria Marta’ intact peel to 6.9% of TAP (Figure 3B). In the intact peel of the cultivar ‘Redhaven’, the average content of total ANTs was lower, but statistical analysis showed no difference (*p* > 0.05) between the tissues. Cyanidin-3-glucoside was the most abundant ANT representative in the control peach peel. The share of TAP in the control peel of the cultivar ‘Maria Marta’ was 19.6% of TAP, and that of ‘Redhaven’ was 22.8% of TAP (Figure S2). The second ANT representative in peach peel was cyanidin-3-rutinoside, present in both cultivars (Table 3). In ‘Redhaven’, different tissues did not differ in Cy-3-glu and also in Cy-3-rut content (*p* > 0.05), although mean values in the intact tissue indicate that the contents of individual ANT representatives were lower (Table 3). In contrast, ANOVA showed differences in ‘Maria Marta’, which had a significantly (*p* < 0.05) lower content of both representatives in the intact tissue. Cy-3-rut content was 4.7-fold lower compared with the control tissue and 4.2-fold lower compared with the injured tissue (Table 3).

Naringenin hex 1 (FLN) and phloridzin (DHC) were the only representatives of the respective group and were increased only (*p* < 0.05) in the injured peel of the damaged cultivar ‘Redhaven’, while their content in ‘Maria Marta’ did not differ between the tissues.

### 2.5. Principal Component Analysis (PCA) for Peach Flesh

**Figure 4:**
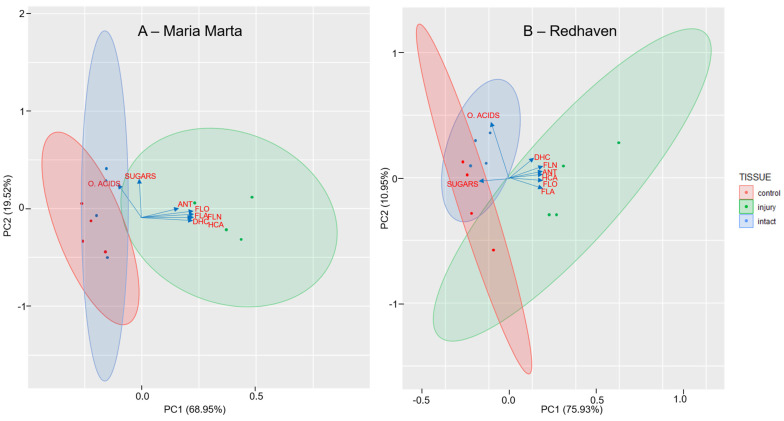
Biplot corresponding to PCA for peach pulp of different tissues according to brown marmorated stinkbug damage—control (tissue from undamaged peaches), intact (undamaged tissue from damaged peaches), and injured (tissue from damaged peaches, from the injury zone in the surface depression). Two peach cultivars are presented—‘Maria Marta’ (**A**) and ‘Redhaven’ (**B**). FLO, flavonols; FLA, flavanols; HCA, hydroxycinnamic acids; DHC, dihydrochalcones; FLN, flavanones, ANT, anthocyanins.

To obtain a comprehensive picture of the response of the flesh to BMSB feeding, two PCA analyses were performed with samples of different types of flesh (control, intact, and injured), the main primary metabolites (organic acids and sugars), and phenolic groups (FLA, FLO, HCA, DHC, FLN, and ANT). A biplot was created to visualise the PCA results (Figure 4A,B). In the first PCA analysis (Figure 4A), cultivar ‘Maria Marta’ was considered, with the first and second components of the PCA model accounting for 68.95% and 19.52% of the total variance, respectively. Figure 4B shows the PCA analysis for ‘Redhaven’, where PC1 accounted for 75.93% and PC2 accounted for 10.95%. For both cultivars, PCA analysis showed that the injured tissue responded to BMSB attack with a higher synthesis of phenolic compounds and had a lower content of primary metabolites (sugars and organic acids) compared to the intact and control tissues.

## 3. Discussion

Brown marmorated stink bug (BMSB) injuries on the peach cultivars ‘Maria Marta’ and ‘Redhaven’ were in the forms of 0.8–9.5 cm surface depressions; a distorted, dented, and discoloured appearance; and red flesh beneath the surface (Figure 1C–F), which is in agreement with Rot et al. (2018) and Acebes-Doria et al. (2016) [6,11]. In addition, more internal damages were present, which reached 0.5–1.8 cm deep into peach flesh. Acebes-Doria et al. (2016) and Joseph et al. (2015) [11,16] also reported a greater extent of internal injuries in peaches. Both internal and external damages were largely present on the sun-exposed site of peach fruits. After dividing peach fruits at harvest into classes according to the size of the damage caused by BMSB in the form of surface depressions, the cultivar ‘Maria Marta’ had 75% of the fruits in the classes, with less than 50% surface depressions per fruit caused by BMSB. In contrast, in the cultivar ‘Redhaven’, more than half (66.7%) of the fruits were classified as having damage, and more than 50% of the fruits had an injured surface. The influence of a cultivar on BMSB preference cannot be evaluated here since the cultivars were not grown at the same location. This unanswered question insinuates what could be done in further research. Nevertheless, both cultivars were included to show their metabolic response to BMSB feeding.

Total sugar, sucrose, and sorbitol contents were lower in the injured tissue of the variety ‘Maria Marta’, while in ‘Redhaven’, the content did not differ between different tissues. Similar findings were reported by Wiman et al. (2015) and Zhou et al. (2016) [8,25] for blueberries and Schumm et al. (2020) [7] for tart cherries. Sugars are the main substrate for the synthesis of phenolic compounds [26], which increased in contrast to sugars in the injured flesh of the cultivar ‘Maria Marta (Table 2). The decreased sugar content (Table 1) in the injured flesh could be a result of increased synthesis of phenolic compounds, which is a known response to protect plants from herbivores [19,27,28]. Since the mentioned mechanism was not proven for ‘Redhaven’, it cannot be stated with certainty as the only reason for the reduction of sugar content in the flesh. BMSB had no effect on the content of total acids in the flesh, whereas individually analysed quinic and shikimic acids in the injured flesh increased in the cultivar ‘Redhaven’ (Table 1). The purpose of the shikimic acid accumulation in the area of the BMSB-induced injury could be to increase the substrate for the synthesis of phenolic compounds, as a wide range of secondary metabolites are derived from shikimic acid [29] in the shikimate pathway, where precursors for aromatic molecules are provided, which are also crucial for the synthesis of flavonoids [30,31]. Quinic acid is involved in the synthesis of caffeoylquinic acids [32,33], which were significantly increased in the injured flesh (Table 2) of the cultivar ‘Redhaven’. This could be an explanation for an increased accumulation of quinic acid in the injury zone.

In general, there was a strong phenolic response to BMSB infestation, especially in the peach flesh in the injury zone. In the cultivar ‘Maria Marta’, flavanols (FLA) content increased by 2.4-fold, hydroxycinnamic acids (HCA) content increased by 5.0-fold, flavonols (FLO) content increased by 3.2-fold, flavanonens (FLN) content increased by 11.3-fold, and dihydrochalcones (DHC) content (only phloridzin was analysed) increased by 3.2-fold compared with the control (Table 2). Interestingly, a representative of anthocyanins (ANT)—cyanidin-3-glucoside—was also analysed in the injured zone of the peach flesh of both cultivars, which is not usually reported in peach flesh [34]. Cyanidin-3-glucoside was not detected in the intact tissue of the control with mass spectrometry. Similarly, phenolics in the injured flesh also increased in ‘Redhaven’: FLA increased by 8.1-fold, HCA increased by 6.3-fold, FLO increased by 5.3-fold, FLN increased by 5.3-fold, and DHC increased by 4.1-fold compared with the control. Consequently, TAP also increased 3.3-fold in ‘Maria Marta’ and 6.9-fold in ‘Redhaven’ in the injured flesh of the fruits damaged by BMSB, compared with the values in the control tissue (Table 2). Ivancic et al. (2022) [35] also reported the same response in olives. Plants respond to a pest attack with increased synthesis of phenolic compounds to reduce or destroy the palatability of the plant in which they are produced [23]. Mostly, these are compounds that act directly as repellents, deterrents, and antidigestants or indirectly as attractants to natural enemies [19,36]. A phenolic response to chewing herbivores occurs through the jasmonic acid signalling pathway [37], which is induced by jasmonate-inducible genes and triggered by the sheath saliva of BMSB [8,38]. After recognising the elicitors produced by herbivores in their oral secretions, plants are able to activate signalling molecules, such as jasmonic acid, which translocates from one part of the injury to other parts of the plant to induce various transcription factors that trigger different gene expressions [39]. Among them, jasmonic acid translocates into the plastides and chloroplasts to activate phenylalanine ammonia lyase (PAL) [40], which is the key enzyme of the phenylpropanoid shikimate pathway and transforms phenylalanine into different phenolic compounds [41].

Destruction of plant cells by stink bug feeding may also act as an elicitor for increased phenolic synthesis, as this leads to the release of proteinases that degrade proteins into the aromatic amino acids of phenylalanine, tyrosine, and tryptophan [42]. The phenolic response in peach flesh injured by BMSB was present only in the zone of the puncture wounds (the injured), as the content of almost all phenolics in the intact tissue was not different from that of the control. An exception in this study was phloridzin, which also increased in the intact tissue of ‘Maria Marta’ (Table 2). The response of the injured flesh to BMSB attack is well summarised in the PCA analysis (Figure 4A,B), which shows that the synthesis of phenolic compounds is increased and the content of primary metabolites (sugars and organic acids) is increased compared to intact and control tissues.

The phenolic response to a BMSB attack was clear with FLA representatives procyanidin dimers, procyanidin trimers, and catechin, where the last increased by 9.9-fold in the injury flesh of the cultivar ‘Maria Marta’ and increased by 10.2-fold in ‘Redhaven’ compared with the control tissue (Table 2). Catechin was previously related to plant–herbivore interactions [43,44]. HCA representatives also responded to BMSB suction similarly in both cultivars, with chlorogenic acid and 3-feruloylquinic acid showing higher levels compared with the control tissue. Chlorogenic acid has been shown to be an efficient defence molecule against a wide range of insect herbivores [32,45,46,47], increasing 9.9-fold in injured flesh in ‘Maria Marta’ and 10.2-fold in ‘Redhaven’ (Table 2) compared with the control tissue. Defence mechanisms involving chlorogenic acid are species-specific to both the plant and the insect [23,46,47]. Lee et al. (2017) [47] reported that high levels of chlorogenic acid in the pith of *Nicotiana attenuata* Steyd. reduce the growth of larvae of the stem borer *Trichobaris mucorea* G.C.Champion, 1909, but, on the other hand, have little effect on the attack of leaf-chewing or sucking insects. It may also reduce the activity of digestive enzymes in insect guts by oxidising chlorogenic acid to chlorogenoquinones [48].

In peach peels, the phenolic response was weaker, especially in the ‘Maria Marta’ cultivar. In ‘Redhaven’, FLA, FLO, FLN (naringenin hexoside analysed only), and DHC (phloridzin analysed only) responded to a BMSB attack in the injured peel with an increase in total content and most individual representatives of the associated phenolic group compared with the control. The content of TAP in the peels of the damaged fruits of ‘Maria Marta’ did not differ between the injured and the intact peels. On the other hand, the content of TAP in the cultivar ‘‘Redhaven’ was 1.3-fold higher in the injured peel than in the control (Table 3). As aforementioned, it was very difficult to visually limit the area of the BMSB puncture on the damaged foetus because individual surface depressions merged (Figure 1D). Thus, parts of the intact tissue were presumably sampled as well. In the ‘Redhaven’ cultivar, the content of individual representatives of HCA, FLA, FLO, and DHC in some cases did not differ from that of the injured tissue examined, which may confirm the assumption that too much surface depression was sampled. In addition, ‘Maria Marta’ was less damaged in terms of the degree of damage to the fruit (Figure 2), which probably also influenced the weak phenolic response of the peel. Compared with the results for the flesh (Table 2), unexpected results for the ANT content of the peel tissues were obtained for the cultivar ‘Maria Marta’. The two representatives of ANT, cyanidin-3-glucoside and cyanidin-3-rutinoside, and consequently the total content of ANT in the intact peel of BMSB-damaged peaches, were lower compared with the control and the damaged peels. The surface depression caused by BMSB was concentrated on the sun-exposed part of the fruit according to a visual assessment of the fruit, which might be related to the desire of BMSB for red-coloured fruit [49]. In contrast to the intact peel of BMSB-damaged fruits, an area of undamaged peel that included the shaded side of the peel was sampled, which, as reported by Gacnik et al. (2021) and Zupan et al. (2014) [50,51], had a less intensive red fruit colour. However, since the colour of the fruit was not the subject of the experiment, this cannot be stated with certainty.

## 4. Materials and Methods

### 4.1. Plant Material

For this study, brown marmorated stink bug (BMSB)-damaged and undamaged peaches at the technological ripening stage of the cultivar ‘Maria Marta’ (MM) were collected from the orchard of the Horticultural Centre of the Biotechnical Faculty, Orehovlje, near Nova Gorica (45°53′28″ N, 13°35′42″ E) and the cultivar ‘Redhaven’ (RH) from the orchard in Drnovk (46°0′16.7184″ N, 13°31′21.0072″ E), as both cultivars were severely damaged by BMSB infestation in 2019. The trees in both orchards are 10 years old and grow on flysch soils (alternating layers of sand, sandstone, and marl) with a sub-Mediterranean climate. At the time of stone hardening (6 June 2019), damaged fruits with characteristic symptoms—gummosis (Figure 1B)—were visually marked for later sampling at harvest based on the reports by Rot et al. (2018) and Acebes-Doria et al. (2016) [6,11]. The trees were in their growing season, and the analyses were conducted according to the integrated cultivation system and with the same technological procedures.

Six peach trees were selected based on similar growth vigour for each cultivar, and 30 BMSB-damaged peaches and 30 undamaged peaches were collected from the middle branches of the trees that had similar sun exposure and grew in the same direction (two damaged and two undamaged fruits from one tree). BMSB and typical damages were identified based on the reports of Rot et al. (2018) and Acebes-Doria et al. (2016) [6,11]. Injuries appeared as superficial depressions with a distorted, dented, and discoloured appearance and red flesh below the surface. The fruits of the cultivar ‘Redhaven’ were collected during the harvest period on 22 July, and those of the cultivar ‘Maria Marta’ were collected on 29 July 2019.

On each of the fruits injured by BMSB, the damage degree was visually assessed (surface impressions) by evaluating the injury zones on four levels depending on the fruit surface: <25%, 25–50%, 50–75%, and >75% injury zones per fruit. Each surface depression was measured on the surface of the peach with the peel, on the surface under the peel (peel removal, Figure 1E), and at the depth of the injury in the flesh. Damage zones were determined by averaging the depression diameter (cm) measurements towards fruit width and fruit height.

### 4.2. Sampling

On the harvest day, BMSB-damaged and undamaged peaches in technological maturity were taken to the laboratory, where the tissues (flesh and peel) were separated from the fruit for the analysis of sugars, organic acids, and phenolics. Damage was visually detected as surface depressions with discoloration on the peel and a red discoloration of the peach flesh. The plant material was cut out with a 10 mm biopsy punch (or less if the depression was smaller) as accurately as possible to a depth of 3 mm, encompassing the peel and flesh. The peel was then carefully separated from the flesh by peeling. Peel and flesh were collected from peaches not damaged by BMSB (the control). BMSB-damaged peaches were separated into two parts. From the undamaged part of the BMSB fruit, the intact peel and flesh were separated (intact). The second part was the peel and the flesh directly in the injury zone—depression area injured). All the materials were immediately frozen in liquid nitrogen.

### 4.3. Sugar and Organic Acid Content Analysis

Individual sugars and organic acids were analysed in the peach flesh. For this, 1 g of combined flesh from five peaches was extracted with 5 mL of double-distilled water. The compounds were extracted on an orbital shaker (Heidolph, Schwabach, Germany, Unimax 1010) for 30 min and then centrifuged on an Eppendorf Centrifuge 5810 R (Thermo Fisher Scientific, Vantaa, Finland) at 10,000 rpm for 7 min at 4 °C. The supernatant was then filtered through a 0.25 μm cellulose filter (Chromafil A-25/25; Macherey-Nagel, Düren, Germany) and stored in vials at −20 °C until analysis with the Thermo Finnigan Surveyor HPLC system (Thermo Scientific, San Jose, CA, USA).

HPLC conditions and equipment for both analyses were based on Gačnik et al. (2021) [52]. They are shown in Table S1. Identification of sugars (fructose, sucrose, and glucose) and organic acids (citric, malic, ascorbic, fumaric, and shikimic acids) was held with the use of external standards (Sigma Aldrich, St. Louis, MO, USA). The contents were calculated and expressed in g/kg fresh weight (FW). The results for quinic, fumaric, and shikimic acids were expressed as mg/kg FW.

### 4.4. Analysis of Individual Phenolic Compounds

For the analysis of individual phenolic compounds in the peel, 0.5 g and in the flesh, 2 g of previously homogenised tissue collected from 5 peaches were weighed into a centrifuge tube and poured over 1.5 mL (peel) or 2 mL (flesh) of extraction solution, which was a mixture of 80% methanol, 3% formic acid, and double-distilled water. Extraction was performed in a cooled ultrasonic bath (Sonis 3, Iskra PIO, Šentjernej, Slovenia) at 2 °C for 1 h. The samples were then centrifuged at 10,000 rpm for 7 min at 4 °C and filtered into vials using 0.20 µm polyamide filters (Chromafil AO-20/25 polyamide filters; Macherey-Nagel, Germany).

Quantification of individual phenolics was performed by high-performance liquid chromatography (HPLC; Thermo Scientific; San Jose, CA, USA) with a DAD detector set to 280, 350, and 530 nm wavelengths. Protocols were performed as described by Gačnik et al. (2021) [52]. The HPLC conditions and equipment used for the analysis of phenolic compounds are shown in Table S1. Table S2 lists the gradient according to which the samples were eluted.

Identification of phenolic compounds in the peach peel and the pulp was made by comparing their retention times with standards using mass spectrometry (LTQ XLTM Linear Ion Trap mass spectrometer; Thermo Scientific, Waltham, MA, USA) and electrospray ionisation. The scans were performed from *m*/*z* 115 to 1500, operating in the negative ion mode for all phenolic groups, with the exception of the anthocyanins (ANT), operating in the positive ion mode. MS conditions, based on Mikulic-Petkovsek et al. (2013) [53], are represented in Table S1.

For quantification, the following standards were used. Flavanols (FLA): procyanidin B1, catechin, and epicatechin from Fluka Chemie (Buchs, Sankt. Gallen, Swiss); hydroxycinnamic acids (HCA): *p*-coumaric acid, caffeic acid, and neochlorogenic acid were obtained from Fluka Chemie, and chlorogenic acid and cryptochlorogenic acid were obtained from Sigma-Aldrich. Flavonols (FLO): quercetin-3-rutinoside, quercetin-3-galactoside, quercetin-3-glucoside, quercetin-3-xyloside, quercetin-3-glucuronide, and kaempferol-3-glucoside were obtained from Fluka Chemie; quercetin-3-arabinopyranoside and quercetin-3-arabinofuranoside were obtained from Apin Chemicals Ltd. (Compton, Newbury, Berkshire, UK); and isorhamnetin-3-glucoside from Extrasynthèse (Genay, France) and quercetin-3-rhamnoside were obtained from Sigma-Aldrich. Dihydrochalcones (DHC): phloridzin were obtained from Fluka Chemie. Flavanones (FLN): naringenin were obtained from Sigma-Aldrich, and ANT: cyanidin-3-glucoside were obtained from Sigma-Aldrich.

The contents of phenolic compounds were calculated from the peak areas of the sample and the corresponding standards and are expressed in mg/kg FW. Total analysed phenolics (TAP) are represented as a sum of all the analysed phenolic compounds in peach fruits. The content of each phenolic group (FLA, HCA, FLO, FLN, and DHC) is represented as a sum of all individual representatives of a singular phenolic group.

### 4.5. Statistical Analysis

R-commander statistical software 4.0.5. (R Formation for Statistical Computing, Auckland, New Zealand) was used to analyse the data. Differences between differently damaged flesh and peel tissues (control, intact, and injured) were determined with a one-way ANOVA and Tukey’s test with 95% confidence for each of the two peach cultivars separately. The assumption of normality by the analysis of model residuals was checked using the QQ plot and the Shapiro–Wilk normality test. The homogeneity of variance was checked with Levene’s test.

Principal component analysis (PCA) was performed for the determination of the contents of primary metabolites (sugars and organic acids) and main phenolic groups (flavonols, flavanols, hydroxycinnamic acids, flavanones, dihydrochalcones and anthocyanins) in the peach flesh differently damaged tissues by BMSB for two peach cultivars (‘Maria Marta’ and ‘Redhaven’) separately.

## 5. Conclusions

The brown marmorated stink bug (BMSB) is a highly destructive and polyphagous invasive pest that poses a serious threat to more than 100 reported host plants. This research is the first to detail how peach peel and flesh from two cultivars—‘Maria Marta’ and ‘Redhaven’—responded to BMSB feeding at the metabolic level. The total sugar, sucrose, and sorbitol contents were reduced in the injured tissue of the cultivar ‘Maria Marta’, whereas in ‘Redhaven’, the contents did not differ between different tissues. In general, a strong phenolic response to BMSB infestation was evident in peach flesh in the injured zone; in the cultivar ‘Maria Marta’, the content of flavanols increased by 2.4-fold, the content of hydroxycinnamic acids increased by 5.0-fold, the content of flavonols increased by 3.2-fold, the content of flavanones increased by 11.3-fold, and the content of dihydrochalcones (only phloridzin was analysed) increased by 3.2-fold compared with the control. The response of ‘Redhaven’ was even stronger. Consequently, the total phenolic content in the injured flesh also increased, 3.3-fold in ‘Maria Marta’ and 6.9-fold in ‘Redhaven’, compared with the control. Feeding of BMSB not only induced the synthesis of cyanidin-3-glucoside, which is usually not found in peach pulp, but also increased the content of almost all individual representatives, especially the flavanols—procyanidin dimers, procyanidin trimers, and catechin. The last was present in the injured pulp of the cultivar ‘Maria Marta’; it was increased by 9.9-fold in ‘Maria Marta’ and by 10.2-fold in ‘Redhaven’ compared with the control tissue, as well as two hydroxycinnamic acids—chlorogenic acid and 3-feruloylquinic acid. In comparison, in the peel of the examined peaches, the phenolic response was weaker, especially in the ‘Maria Marta’ cultivar. The study showed that peaches of both cultivars responded to BMSB attack with phenolics restricted to the area of damage, confirming previously reported results in blueberries (Zhou et al., 2016 [8]) and olives (Ivancic et al., 2022 [35]). It should be mentioned that this study is limited to the area of recent discovery of BMSB occurrence, and it is not clear how the plant adapts at a metabolic level to the pest in its perennial presence. It also remains an open question whether BMSB prefers different peach cultivars. Studies on the metabolic response of plants to the pest are certainly important, especially for further investigations on specific plant defence mechanisms, and they also provide plant breeders with information on useful traits. This type of research can also serve as a basis for the development of new methods for obtaining potential chemicals for environmentally acceptable plant protection measures.

## Figures and Tables

**Table 1:** Sugar and organic acid content (g/kg FW; * mg/kg FW; mean ± SE) in peaches damaged by a brown marmorated stink bug.

	‘Maria Marta’			‘Redhaven’		
	Control	Intact	Injury	Control	Intact	Injury
Sucrose	92.11 ± 5.60 b	81.34 ± 2.84 b	48.92 ± 0.82 a	72.11 ± 7.46 a	100.6 ± 14.64 a	86.14 ± 7.07 a
Glucose	9.57 ± 1.27 a	9.88 ± 0.33 a	10.54 ± 0.2 a	13.02 ± 1.39 a	12.59 ± 0.25 a	11.65 ± 0.42 a
Fructose	11.38 ± 0.57 a	10.88 ± 0.47 a	12.18 ± 0.31 a	13.85 ± 1.52 a	13.14 ± 0.45 a	13.25 ± 0.4 a
Sorbitol	2.03 ± 0.15 b	1.64 ± 0.20 b	0.96 ± 0.02 a	0.94 ± 0.32 a	1.20 ± 0.12 a	0.92 ± 0.30 a
**Total Sugars**	**114.9 ± 9.11 b**	**103.7 ± 2.07 b**	**72.6 ± 1.09 a**	**99.92 ± 5.23 a**	**127.5 ± 14.16 a**	**112.0 ± 7.52 a**
Citric	3.55 ± 0.21 a	3.69 ± 0.11 a	3.12 ± 0.17 a	4.60 ± 1.02 a	3.73 ± 0.46 a	3.36 ± 0.17 a
Malic	3.96 ± 0.78 a	4.95 ± 0.27 a	3.16 ± 0.40 a	5.39 ± 0.63 a	6.38 ± 1.32 a	4.36 ± 0.40 a
Quinic *	86.92 ± 7.12 a	100.8 ± 4.39 a	121.2 ± 55.13 a	60.06 ± 11.7 a	113.2 ± 19.95 a	221.1 ± 9.28 b
Shikimic *	0.21 ± 0.01 a	0.35 ± 0.05 a	0.76 ± 0.42 a	0.35 ± 0.09 a	0.35 ± 0.05 a	1.44 ± 0.10 b
Fumaric *	12.51 ± 1.84 a	10.58 ± 1.07 a	24.47 ± 9.93 a	10.97 ± 1.30 a	11.08 ± 1.22 a	11.38 ± 0.16 a
**Total Acids**	**7.62 ± 0.89 a**	**8.74 ± 0.3 a**	**6.43 ± 0.57 a**	**10.06 ± 1.6 a**	**10.23 ± 1.78 a**	**7.95 ± 0.56 a**

Legend: control—undamaged tissue from undamaged peaches, intact—undamaged tissue from damaged peaches, and injured—damaged tissue. Data are means ± standard error. Letters (a and b) in rows show significant differences between tissues, separately for each cultivar, obtained by analysis of variance (ANOVA) and post hoc analysis by Duncan’s multiple range test at 95% confidence.

**Table 2:** Content of individual phenolic compounds and phenolic groups (mg/kg FW; mean ± SE) in ‘Maria Marta’ and ‘Redhaven’ peach flesh damaged by a brown marmorated stink bug.

	‘Maria Marta’			‘Redhaven’		
	Control	Intact	Injured	Control	Intact	Injured
3-CQA (neochlorogenic acid)	9.79 ± 3.68 a	8.97 ± 0.94 a	23.61 ± 3.63 b	24.59 ± 5.72 a	30.7 ± 0.94 a	75.1 ± 10.15 b
Caffeic acid hex 1	0.44 ± 0.06 a	0.14 ± 0.04 a	1.35 ± 0.29 b	0.30 ± 0.05 a	0.40 ± 0.07 a	3.02 ± 0.47 b
Caffeic acid hex 2	0.06 ± 0.01 a	0.04 ± 0.01 a	0.20 ± 0.03 b	0.03 ± 0.01 a	0.06 ± 0.01 a	0.26 ± 0.04 b
*p*-CoA hex 1	0.54 ± 0.07 a	0.17 ± 0.05 a	1.65 ± 0.36 b	0.37 ± 0.07 a	0.49 ± 0.08 a	3.70 ± 0.58 b
*p*-CoA hex 2	0.03 ± 0.00 a	0.02 ± 0.00 a	0.10 ± 0.01 b	0.02 ± 0.00 a	0.03 ± 0.01 a	0.13 ± 0.02 b
3-*p*-CoQA	0.47 ± 0.06 a	0.15 ± 0.05 a	1.45 ± 0.32 b	0.32 ± 0.06 a	0.43 ± 0.07 a	3.25 ± 0.51 b
5-CQA1 (chlorogenic acid)	15.2 ± 3.04 a	16.84 ± 1.27 a	150.5 ± 36.93 b	23.17 ± 7.99 a	31.7 ± 2.17 a	237.1 ± 30.89 b
5-CQA2	9.46 ± 1.05 a	9.28 ± 1.00 a	28.04 ± 2.11 b	12.16 ± 2.1 a	19.05 ± 4.00 a	77.25 ± 1.78 b
3-FQA1	0.11 ± 0.02 a	0.13 ± 0.01 a	1.13 ± 0.28 b	0.17 ± 0.06 a	0.24 ± 0.02 a	1.78 ± 0.23 b
4-CQA	3.73 ± 0.3 a	2.21 ± 0.32 a	6.52 ± 0.59 b	2.65 ± 0.66 a	3.60 ± 0.41 a	10.42 ± 0.28 b
5-*p*-CoQA 1	0.60 ± 0.11 a	0.15 ± 0.03 b	0.54 ± 0.08 a	0.18 ± 0.04 a	0.21 ± 0.07 a	0.84 ± 0.04 b
5-*p*-CoQA 2	0.25 ± 0.02 a	0.05 ± 0.01 b	0.17 ± 0.03 a	0.06 ± 0.02 a	0.07 ± 0.01 a	0.59 ± 0.20 a
diCQA1	0.30 ± 0.04 a	0.62 ± 0.13 a	3.36 ± 0.84 b	0.84 ± 0.15 a	1.50 ± 0.24 a	3.96 ± 0.32 b
diCQA2	0.22 ± 0.03 a	0.46 ± 0.10 a	2.52 ± 0.63 b	0.63 ± 0.11 a	1.13 ± 0.18 a	2.97 ± 0.24 b
diCQA3	1.17 ± 0.12 a	2.75 ± 0.62 b	3.24 ± 0.14 b	4.21 ± 0.79 a	7.08 ± 1.11 a	17.53 ± 1.62 b
Sinapoyl hex	0.27 ± 0.03 a	0.32 ± 0.05 a	1.85 ± 0.25 b	0.33 ± 0.15 a	0.45 ± 0.09 a	2.78 ± 0.32 b
**HCA ^1^**	**45.49 ± 8.98 a**	**42.63 ± 3.21 a**	**228.9 ± 43.52 b**	**70.76 ± 17.19 a**	**98.25 ± 8.68 a**	**444.6 ± 31.21 b**
Procy dimer 1	26.1 ± 5.55 a	3.76 ± 1.38 a	65.02 ± 14.71 b	6.13 ± 1.17 a	10.31 ± 1.2 a	111.0 ± 17.22 b
procy dimer 2	6.40 ± 0.82 a	2.04 ± 0.62 a	19.76 ± 4.29 b	4.40 ± 0.80 a	5.82 ± 1.00 a	44.19 ± 6.88 b
Procy dimer 3	0.99 ± 0.15 a	0.61 ± 0.11 a	3.17 ± 0.46 b	0.53 ± 0.10 a	0.93 ± 0.19 a	4.23 ± 0.57 b
Procy dimer 4	28.03 ± 3.91 a	10.46 ± 1.13 b	43.63 ± 6.48 a	10.42 ± 3.47 a	14.04 ± 3.05 a	53.97 ± 5.30 b
Catechin	3.17 ± 0.63 a	3.52 ± 0.27 a	31.42 ± 7.71 b	4.84 ± 1.67 a	6.62 ± 0.45 a	49.52 ± 6.45 b
Procy trimer 1	2.85 ± 0.57 a	3.16 ± 0.24 a	28.19 ± 6.92 b	4.34 ± 1.50 a	5.94 ± 0.41 a	44.43 ± 5.79 b
Procy trimer 2	7.89 ± 0.64 a	4.68 ± 0.68 a	13.8 ± 1.25 b	5.60 ± 1.40 a	7.61 ± 0.86 a	22.08 ± 0.59 b
Procy trimer 3	12.55 ± 2.49 a	2.98 ± 0.64 b	12.02 ± 1.28 a	3.44 ± 1.01 a	5.51 ± 1.77 a	19.98 ± 2.75 b
Procy tetramer	9.87 ± 0.80 a	5.86 ± 0.85 a	17.25 ± 1.56 b	7.00 ± 1.75 a	9.52 ± 1.07 a	27.59 ± 0.74 b
**FLA ^1^**	**97.85 ± 15.42 a**	**37.06 ± 5.19 a**	**234.3 ± 39.07 b**	**46.7 ± 12.03 a**	**66.3 ± 9.57 a**	**377 ± 35.44 b**
Roseoside	6.80 ± 1.03 a	4.23 ± 0.76 a	21.84 ± 3.20 b	3.66 ± 0.70 a	6.41 ± 1.34 a	29.12 ± 3.93 b
Q-3-rut	0.06 ± 0.01 a	0.08 ± 0.03 a	1.17 ± 0.37 b	0.13 ± 0.02 a	0.15 ± 0.04 a	2.46 ± 0.67 b
Q-3-gal	8.00 ± 2.08 a	14.49 ± 2.81 ab	22.26 ± 1.02 b	12.16 ± 2.92 a	19.26 ± 3.38 a	48.86 ± 3.88 b
Q-3-glu	0.68 ± 0.18 a	1.17 ± 0.24 ab	1.70 ± 0.17 b	0.96 ± 0.21 a	1.71 ± 0.34 a	4.29 ± 0.26 b
Kaem-3-rut	0.16 ± 0.06 a	0.40 ± 0.06 a	1.59 ± 0.27 b	0.34 ± 0.02 a	0.57 ± 0.05 a	5.41 ± 1.28 b
Iso-3-rut	0.86 ± 0.17 ab	0.68 ± 0.08 b	1.41 ± 0.16 a	1.16 ± 0.41 a	1.78 ± 0.49 a	3.73 ± 0.36 b
Q-3-xyl	0.03 ± 0.01 ab	0.03 ± 0.00 b	0.05 ± 0.01 a	0.04 ± 0.02 a	0.07 ± 0.02 a	0.14 ± 0.01 b
Kaem hex 1	0.05 ± 0.01 a	0.06 ± 0.01 a	0.32 ± 0.04 b	0.06 ± 0.03 a	0.08 ± 0.02 a	0.49 ± 0.06 b
Kaem hex 2	0.12 ± 0.05 a	0.20 ± 0.02 a	1.02 ± 0.16 b	0.24 ± 0.07 a	0.13 ± 0.03 a	2.51 ± 0.17 b
Iso-hex 1	0.14 ± 0.05 a	0.23 ± 0.02 a	1.18 ± 0.18 b	0.27 ± 0.08 a	0.15 ± 0.03 a	2.89 ± 0.20 b
Iso-hex 2	0.03 ± 0.00 a	0.07 ± 0.01 a	0.39 ± 0.10 b	0.10 ± 0.02 a	0.17 ± 0.03 a	0.46 ± 0.04 b
Q-3-arafur	in traces	in traces	in traces	in traces	in traces	in traces
Q-3-rham	0.03 ± 0.00 a	0.06 ± 0.01 a	0.32 ± 0.08 b	0.08 ± 0.01 a	0.14 ± 0.02 a	0.38 ± 0.03 b
Kaem acetyl hex	in traces	in traces	in traces	in traces	in traces	in traces
Q-3-rhamnosyl hex 1	in traces	in traces	in traces	in traces	in traces	in traces
Q-3-rhamnosyl hex 2	in traces	in traces	in traces	in traces	in traces	in traces
Q-3-arapyr	in traces	in traces	in traces	in traces	in traces	in traces
**FLO ^1^**	**16.98 ± 3.45 a**	**21.72 ± 4.02 a**	**53.41 ± 5.17 b**	**19.24 ± 4.43 a**	**30.65 ± 5.67 a**	**101 ± 9.4 b**
Nar hex 1	0.01 ± 0.00 a	0.02 ± 0.00 a	0.08 ± 0.01 b	0.02 ± 0.00 a	0.03 ± 0.00 a	0.26 ± 0.06 b
Nar hex 2	0.04 ± 0.01 a	0.08 ± 0.02 a	0.42 ± 0.11 b	0.11 ± 0.02 a	0.19 ± 0.03 a	0.50 ± 0.04 b
Nar hex 3	0.07 ± 0.01 a	0.16 ± 0.03 a	0.85 ± 0.21 b	0.21 ± 0.04 a	0.38 ± 0.06 a	1.00 ± 0.08 b
**FLN ^1^**	**0.12 ± 0.02 a**	**0.25 ± 0.05 a**	**1.35 ± 0.33 b**	**0.33 ± 0.06 a**	**0.60 ± 0.09 a**	**1.76 ± 0.13 b**
**Phloridzin (DHC) ^1^**	**0.17 ± 0.03 a**	**0.43 ± 0.08 b**	**0.54 ± 0.03 b**	**0.52 ± 0.08 a**	**0.76 ± 0.05 a**	**2.13 ± 0.09 b**
**Cy-3-glu (ANT) ^1^**	**-**	**-**	**7.09 ± 2.25**	**-**	**-**	**23.24 ± 12.38**
**TAP ^2^**	**160.6 ± 27.83 a**	**102.1 ± 12.28 a**	**525.5 ± 87.6 b**	**137.6 ± 33.44 a**	**196.6 ± 23.82 a**	**949.7 ± 73.4 b**

Legend: control—undamaged tissue from undamaged peaches, intact—undamaged tissue from damaged peaches, and injured—damaged tissue. ^1^ Sum of the contents of all individual phenolic representatives of the phenolic group in peach fruit flesh. ^2^ Sum of the contents of all identified phenolic compounds in peach fruit flesh. Data are means ± standard error. Letters (a and b) in rows show significant differences between tissues, separately for each cultivar, obtained by analysis of variance (ANOVA) and post hoc analysis by Duncan’s multiple range test at 95% confidence. Abbreviations: HCA: hydroxycinnamic acids, FLA: flavanols, FLO: flavonols, FLN: flavanones, DHC: dihydrochalcones, ANT: anthocyanins, TAP: total analysed phenolics, CQA: caffeoylquinic acid, hex: hexoside, *p*-CoA: *p*-coumaric acid, CoQA: coumaroylquinic acid, FQA: feruloylquinic acid, Q: quercetin, rut: rutinoside, gal: galactoside, glu: glucoside, Kaem: kaempferol, Iso: isorhamnetin, xyl: xyloside, arafur: arabinofuranoside, rham: rhamnoside, arapyr: arabinopyranoside, Nar: naringenin, Cy: cyanidin.

**Table 3:** Content of individual phenolic compounds and phenolic groups (mg/kg FW; mean ± SE) in ‘Maria Marta’ and ‘Redhaven’ peach peel damaged by a brown marmorated stink bug.

	‘Maria Marta’			‘Redhaven’		
	Control	Intact	Intact	Control	Injured	Injured
3-CQA (neochlorogenic acid)	153.2 ± 29.99 a	45.92 ± 6.67 b	136.9 ± 23.19 a	528.0 ± 172.4 a	331.7 ± 44.44 a	616.8 ± 130.0 a
Caffeic acid hex 1	0.32 ± 0.05 a	0.47 ± 0.05 a	0.28 ± 0.04 a	0.62 ± 0.12 a	0.93 ± 0.10 ab	1.32 ± 0.20 b
Caffeic acid hex 2	4.95 ± 0.71 a	7.23 ± 0.55 a	5.60 ± 0.67 a	9.39 ± 1.53 a	12.89 ± 1.08 ab	18.98 ± 2.73 b
Caffeic acid hex 3	0.41 ± 0.06 a	0.60 ± 0.04 a	0.46 ± 0.05 a	0.77 ± 0.13 a	1.06 ± 0.09 ab	1.56 ± 0.22 b
*p*-CoA hex 1	0.35 ± 0.06 a	0.50 ± 0.05 a	0.30 ± 0.05 a	0.66 ± 0.12 a	1.00 ± 0.1 ab	1.42 ± 0.22 b
*p*-CoA hex 2	0.21 ± 0.03 a	0.31 ± 0.02 a	0.24 ± 0.03 a	0.41 ± 0.07 a	0.56 ± 0.05 ab	0.82 ± 0.12 b
3-*p*-CQA	0.39 ± 0.07 a	0.57 ± 0.06 a	0.34 ± 0.05 a	0.76 ± 0.14 a	1.14 ± 0.12 ab	1.62 ± 0.25 b
5-CQA1 (chlorogenic acid)	48.88 ± 5.47 a	53.64 ± 4.24 a	38.11 ± 7.13 a	75.18 ± 18.25 a	102.9 ± 5.03 a	103.2 ± 12.38 a
5-CQA2	16.52 ± 1.96 a	25.73 ± 1.3 b	19.48 ± 1.49 ab	32.33 ± 3.87 a	54.96 ± 0.68 b	54.25 ± 4.67 b
3-FQA	0.67 ± 0.08 a	0.74 ± 0.06 a	0.53 ± 0.10 a	1.04 ± 0.25 a	1.42 ± 0.07 a	1.42 ± 0.17 a
4-CQA	4.25 ± 0.57 a	4.00 ± 0.34 a	3.31 ± 0.22 a	5.06 ± 0.91 a	3.41 ± 1.93 a	3.90 ± 2.06 a
4-*p*-CoQA	0.67 ± 0.13 a	0.56 ± 0.11 a	0.58 ± 0.13 a	1.43 ± 0.20 a	1.15 ± 0.27 a	1.85 ± 0.20 a
5-*p*-CoQA 1	0.80 ± 0.24 a	1.54 ± 0.49 a	0.62 ± 0.23 a	2.12 ± 0.28 a	2.07 ± 0.32 a	4.92 ± 1.19 a
5-*p*-CoQA 2	0.95 ± 0.33 a	0.54 ± 0.14 a	0.72 ± 0.15 a	3.41 ± 0.51 a	2.40 ± 0.24 a	7.27 ± 1.39 b
diCQA1	0.12 ± 0.02 a	0.12 ± 0.01 a	0.14 ± 0.02 a	0.31 ± 0.05 a	0.39 ± 0.04 a	0.87 ± 0.06 b
diCQA2	In traces	In traces	In traces	In traces	In traces	In traces
diCQA3	In traces	In traces	In traces	In traces	In traces	In traces
**HCA ^1^**	**232.7 ± 38.03 a**	**142.5 ± 10.82 a**	**207.7 ± 32.54 a**	**661.5 ± 187.16 a**	**518.0 ± 48.7 a**	**820.2 ± 148.5 a**
Procy dimer 1	42.16 ± 5.26 a	29.18 ± 2.21 ab	25.51 ± 3.55 b	74.89 ± 9.47 a	62.41 ± 9.39 a	111.7 ± 7.12 b
Procy dimer 2	16.24 ± 2.72 a	23.41 ± 2.55 a	14.04 ± 2.15 a	31.23 ± 5.83 a	47.04 ± 4.88 ab	66.58 ± 10.29 b
Procy dimer 3	1.07 ± 0.15 a	1.56 ± 0.12 a	1.21 ± 0.14 a	2.02 ± 0.33 a	2.78 ± 0.23 ab	4.09 ± 0.59 b
Procy dimer 4	44.09 ± 9.16 a	55.85 ± 9.05 a	36.64 ± 6.49 a	69.97 ± 6.85 a	73.69 ± 7.04 a	136.8 ± 20.07 b
Procy timer 5	In traces	In traces	In traces	In traces	In traces	In traces
Procy trimer 1	19.19 ± 3.22 a	27.67 ± 3.01 a	16.59 ± 2.54 a	36.91 ± 6.89 a	55.59 ± 5.77 ab	78.68 ± 12.16 b
Procy trimer 2	47.37 ± 5.30 a	51.97 ± 4.11 a	36.93 ± 6.91 a	72.85 ± 17.69 a	99.71 ± 4.87 a	99.96 ± 11.99 a
Procy timer 3	16.23 ± 2.19 a	15.26 ± 1.30 a	12.65 ± 0.85 a	19.31 ± 3.48 a	13.02 ± 7.37 a	14.88 ± 7.85 a
Procy trimer 4	In traces	In traces	In traces	In traces	In traces	In traces
Procy trimer 5	16.25 ± 3.69 a	9.77 ± 3.19 a	10.97 ± 3.04 a	20.29 ± 4.54 a	16.82 ± 2.82 a	34.72 ± 3.34 b
catechin	56.31 ± 6.30 a	61.79 ± 4.88 a	43.91 ± 8.21 a	86.6 ± 21.03 a	118.6 ± 5.79 a	118.8 ± 14.26 a
Procy tetramer	18.10 ± 2.44 a	17.02 ± 1.45 a	14.11 ± 0.94 a	21.54 ± 3.88 a	14.53 ± 8.23 a	16.6 ± 8.76 a
Epicatechin	4.33 ± 0.82 a	3.62 ± 0.71 a	3.75 ± 0.86 a	9.33 ± 1.33 a	7.45 ± 1.74 a	12.03 ± 1.28 a
**FLA ^1^**	**281.3 ± 37.02 a**	**297.1 ± 27.27 a**	**216.3 ± 27.68 a**	**444.9 ± 66.87 a**	**511.6 ± 25.53 ab**	**694.8 ± 75.23 b**
Roseoside	1.29 ± 0.18 a	1.88 ± 0.14 a	1.45 ± 0.17 a	2.44 ± 0.40 a	3.35 ± 0.28 ab	4.93 ± 0.71 b
Q-3-rut	3.58 ± 0.63 a	8.05 ± 0.93 b	4.58 ± 1.75 ab	21.22 ± 4.10 a	21.2 ± 3.51 a	58.79 ± 11.89 b
Q-3-gal	5.00 ± 0.35 a	6.80 ± 0.49 a	11.15 ± 1.39 b	26.92 ± 8.05 a	38.14 ± 2.35 a	60.74 ± 15.59 a
Q-3-glu	4.39 ± 0.24 a	3.75 ± 0.33 a	6.39 ± 1.87 a	38.62 ± 8.46 a	24.82 ± 4.56 a	86.67 ± 25.64 a
Kaem-3-rut	5.28 ± 0.73 a	5.26 ± 0.39 a	2.96 ± 0.43 b	9.87 ± 2.01 a	11.93 ± 1.46 ab	18.5 ± 1.99 b
Q-3-arafur	0.91 ± 0.17 a	2.72 ± 0.21 b	1.17 ± 0.36 a	7.77 ± 1.02 a	7.31 ± 0.15 a	14.95 ± 2.43 b
Q-3-xyl	0.20 ± 0.02 ab	0.23 ± 0.01 b	0.13 ± 0.03 a	0.36 ± 0.05 a	0.44 ± 0.05 ab	0.65 ± 0.07 b
Iso-3-rut	16.01 ± 1.62 ab	17.76 ± 0.97 b	10.42 ± 2.06 a	27.99 ± 4.2 a	34.19 ± 3.93 ab	51.24 ± 5.6 b
Q-3-glucuronide	0.02 ± 0.00 ab	0.03 ± 0.00 b	0.02 ± 0.00 a	0.04 ± 0.01 a	0.05 ± 0.01 ab	0.07 ± 0.01 b
Kaem hex 1	9.34 ± 1.04 a	6.28 ± 0.47 b	4.17 ± 0.60 b	7.74 ± 2.13 a	5.06 ± 1.19 a	4.64 ± 0.99 a
Kaem hex 2	0.79 ± 0.22 a	2.09 ± 0.25 b	1.59 ± 0.26 ab	13.31 ± 1.84 a	7.39 ± 0.89 a	25.96 ± 4.28 b
Q-3-arapyr	0.08 ± 0.02 a	0.07 ± 0.01 a	0.10 ± 0.01 a	0.18 ± 0.03 a	0.19 ± 0.00 a	0.32 ± 0.04 b
Iso hex 1	0.29 ± 0.08 a	0.77 ± 0.09 b	0.58 ± 0.09 ab	4.90 ± 0.68 a	2.72 ± 0.33 a	9.55 ± 1.58 b
Iso hex 2	0.07 ± 0.01 a	0.07 ± 0.01 a	0.08 ± 0.01 a	0.18 ± 0.03 a	0.23 ± 0.02 a	0.51 ± 0.03 b
Q-3-rha	0.24 ± 0.05 a	0.24 ± 0.02 a	0.28 ± 0.04 a	0.62 ± 0.1 a	0.76 ± 0.07 a	1.71 ± 0.11 b
Kaem acetylhex	0.51 ± 0.13 a	0.36 ± 0.07 a	0.40 ± 0.09 a	0.69 ± 0.17 a	1.09 ± 0.06 a	2.57 ± 0.39 b
Iso acetylhex	0.31 ± 0.02 a	0.36 ± 0.05 a	0.37 ± 0.11 a	0.59 ± 0.18 a	0.96 ± 0.07 ab	2.00 ± 0.50 b
Q-rhamnosyl hex 1	0.11 ± 0.03 a	0.13 ± 0.02 a	0.15 ± 0.04 a	0.57 ± 0.13 a	0.48 ± 0.03 a	1.14 ± 0.12 b
Q-rhamnosyl hex 2	0.17 ± 0.03 a	0.31 ± 0.05 a	0.26 ± 0.08 a	1.85 ± 0.28 a	1.30 ± 0.10 a	3.46 ± 0.35 b
Q-rhamnosyl hex 3	In traces	In traces	In traces	In traces	In traces	In traces
**FLO ^1^**	**48.61 ± 4.61 a**	**57.6 ± 3.37 a**	**46.25 ± 8.2 a**	**165.8 ± 28.58 a**	**161.6 ± 15.5 a**	**348.4 ± 69.23 b**
**Nar hex 1 (FLN) ^1^**	**0.01 ± 0.00 a**	**0.01 ± 00.0 a**	**0.01 ± 00.0 a**	**0.02 ± 0.00 a**	**0.03 ± 0.00 a**	**0.06 ± 0.00 b**
Nar hex 2	In traces	In traces	In traces	In traces	In traces	In traces
**Phloridzin (DHC) ^1^**	**0.29 ± 0.08 a**	**0.30 ± 0.03 a**	**0.46 ± 0.07 a**	**0.57 ± 0.19 a**	**1.32 ± 0.12 ab**	**1.50 ± 0.30 b**
Cy-3-glu	147.1 ± 30.54 b	31.37 ± 5.9 a	130.6 ± 23.48 b	345.8 ± 49.29 a	312.7 ± 47.12 a	463.9 ± 33.98 a
Cy-3-rut	26.48 ± 5.50 b	5.65 ± 1.06 a	23.5 ± 4.23 b	62.24 ± 8.87 a	56.29 ± 8.48 a	83.50 ± 6.12 a
**ANT ^1^**	**173.6 ± 36.04 b**	**37.02 ± 6.96 a**	**154.1 ± 27.7 b**	**408.0 ± 58.16 a**	**369.0 ± 55.6 a**	**547.4 ± 34.73 a**
**TAP ^2^**	**736.6 ± 100.5 a**	**534.5 ± 45.08 a**	**624.7 ± 92.98 a**	**1479 ± 271.2**	**1562 ± 137.2**	**2140 ± 126.3**

Legend: control—undamaged tissue from undamaged peaches, intact—undamaged tissue from damaged peaches, and injured—damaged tissue. ^1^ Sum of the contents of all individual phenolic representatives of the phenolic group in peach peel. ^2^ Sum of the contents of all identified phenolic compounds in peach peel. Data are means ± standard error. Letters (a and b) in rows show significant differences between tissues, separately for each cultivar, obtained by analysis of variance (ANOVA) and post hoc analysis by Duncan’s multiple range test at 95% confidence. Abbreviations: HCA: hydroxycinnamic acids, FLA: flavanols, FLO: flavonols, FLN: flavanones, DHC: dihydrochalcones, ANT: anthocyanins, TAP: total analysed phenolics, CQA: caffeoylquinic acid, hex: hexoside, *p*-CoA: *p*-coumaric acid, CoQA: coumaroylqunic acid, FQA: feruloylquinic acid, Q: quercetin, rut: rutinoside, gal: galactoside, glu: glucoside, Kaem: kaempferol, Iso: isorhamnetin, xyl: xyloside, arafur: arabinofuranoside, arapyr: arabinopyranoside, Nar: naringenin, Cy: cyanidin.

## Data Availability

The data presented in this study are available on request from the corresponding author.

## Supplementary Materials

The supporting information can be downloaded at: https://www.mdpi.com/article/10.3390/ijms25010606/s1.
