# Supplementary material for: Metabolic Response of Peach Fruit to Invasive Brown Marmorated Stink Bug (Halyomorpha halys Stål.)’s Infestation
Source: Int J Mol Sci. 2024 Jan 3;25(1):606. doi: 10.3390/ijms25010606 (PMC10778873; doi:10.3390/ijms25010606)
Supplement: Supplementary file 1 [file ijms-25-00606-s001.zip › ijms-2787384-supplementary.pdf]

SUPPLEMENTAL DATA

Table S1: HPLC and MS conditions, based on and Mikulic-Petkovsek et al. (2013).

	HPLC conditions		
	**Organic acids**	**Sugars**	**Phenolics**
**Recorded spectra**	210 nm	/	200–600 nm
**Detector**	UV detector	Refractive index detector (RI)	Diode array detector (DAD)
**Column**	Rezex ROA H + (8%); Phenomenex	Rezex RCM-monosaccharide CA (+2%); Phenomenex	Gemini C18 (150 × 4.6mm; 3 μm); Phenomenex
**Column temperature**	65 °C	65 °C	25 °C
**Injection**	20 μL	20 μL	20 μL
**Flow rate**	0.6 mL/min	0.6 mL/min	0.6 mL/min
**Total run time**	30 min	30 min	45 min
**Mobile phase**	4 mM sulfuric acid	Bidestilled water	A: 0.1% formic acid + 96.9% biH_2O + 3% acetonitrile; B: 0.1% formic acid + 96.9% acetonitrile + 3% biH_2O

	**MS conditions**
**Full scan (*m/z*)**	115–1500
**Injection volume**	10 μL
**Flow rate**	0.6 mL/min
**Heater and capillary temperature**	320 °C
**Sheat-Aux-Sweep Gas Flow Rate**	50 – 20 – 0 arb
**I Spray Voltage (kV)**	3.5
**Capillary voltage (V)**	10
**Mobile phase**	A: 0.1% formic acid + 96.9% biH_2O + 3% acetonitrile; B: 0.1% formic acid + 96.9% acetonitrile + 3% biH_2O

Table S2: HPLC and MS gradient, according to Mikulic-Petkovsek et al. (2013)

**HPLC and MS Gradient**	
**Time (min)**	**B (%)**
0	5
15	20
20	30
25	30
30	90
35	100
45	5
50	5

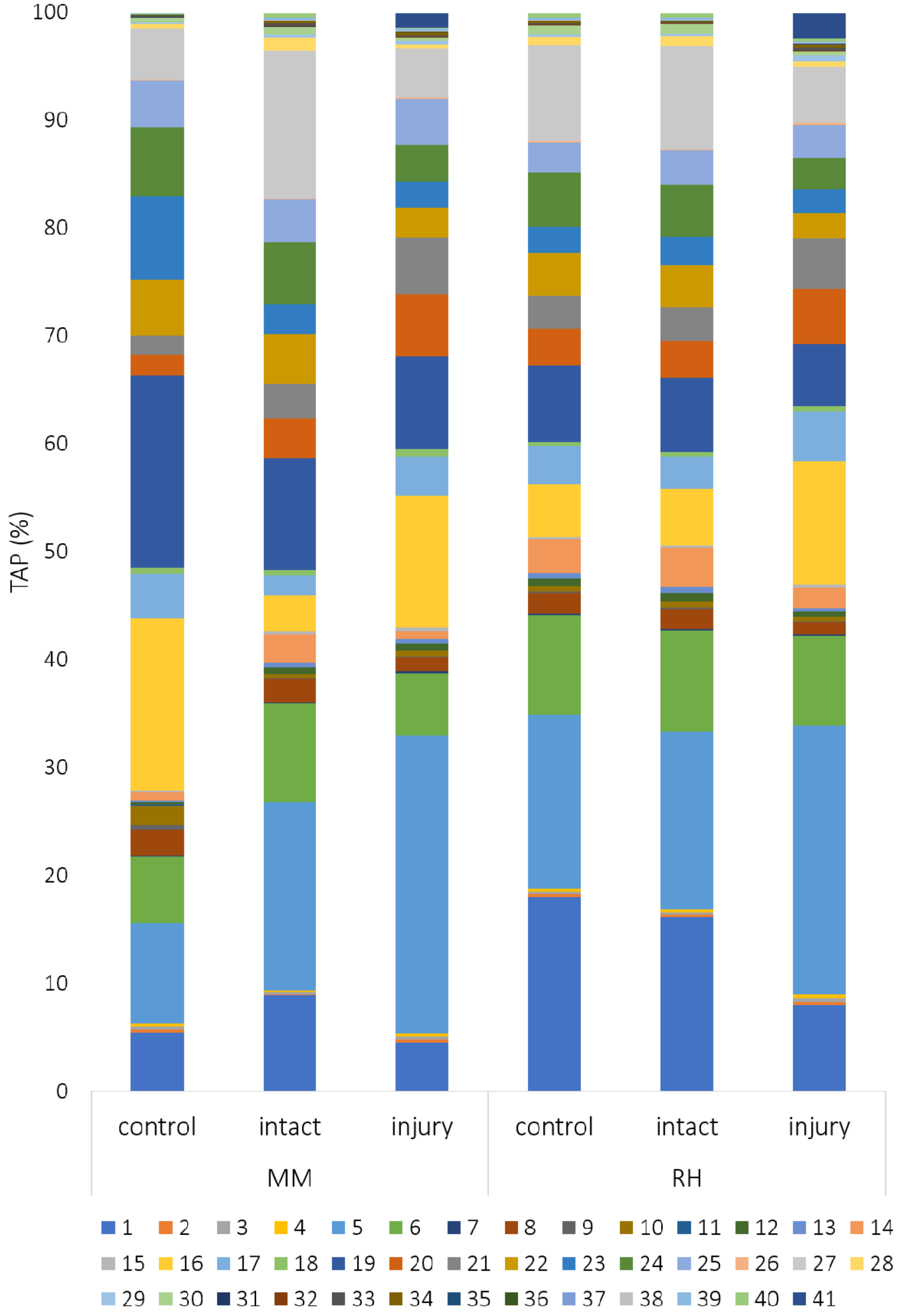

Figure S1: Proportion of each phenolic compound with regard to the total phenolic content (TAP) in the peach flesh of different tissues, depending on the injuries caused by a brown marmorated stink bug

– control (tissue from undamaged peaches), intact (undamaged tissue from damaged peaches) and injured (tissue of damaged peaches, from the injury zone in the surface depression). Two peach cultivars are presented – ‘Maria Marta’ (MM) and ‘Redhaven’ (RH). 1: neochlorogenic acid; 2: caffeic acid hexoside 1; 3: *p*-coumaric acid hexoside 1; 4: 3-*p*-caffeoylquinic acid; 5: chlorogenic acid; 6: 5-caffeoylquinic acid 2; 7: 3-feruloylquinic acid; 8: 4-caffeoylquinic acid; 9: 5-*p*-coumaroyl quinic acid 1; 10: 5-*p*-coumaroylquinic acid 2; 11: 5-*p*-coumaroylquinic acid 3; 12: dicaffeoylquinic acid 1; 13: dicaffeoylquinic acid 2 ; 14: dicaffeoylquinic acid 3; 15: sinapoylhexoside; 16: procyianidin dimer 1; 17: procyanidin dimer 2; 18: procyanidin dimer 3; 19: procyanidin dimer 4; 20: catechin; 21: procyanidin trimer 1; 22: procyanidin trimer 2; 23: procyanidin trimer 3; 24: procyanidin tetramer; 25: roseoside; 26: quercetin-3-rutinoside; 27: quercetin-3-galactoside; 28: quercetin-3-glucoside; 29: kaempferol-3-rutinoside; 30: isorhamnetin-3-rutinoside; 31: quercetin-3-xyloside; 32: kaempferol hexoside 1; 33: kaempferol hexoside 2; 34: isorhamnetin hexoside 1; 35: isorhamnetin hexoside 2; 36: quercetin-3-rhamnoside; 37: naringenin hexoside 1; 38: naringenin hexoside 2; 39: naringenin hexoside 3; 40: phloridzin; 41: cyanidin-3-glucoside.

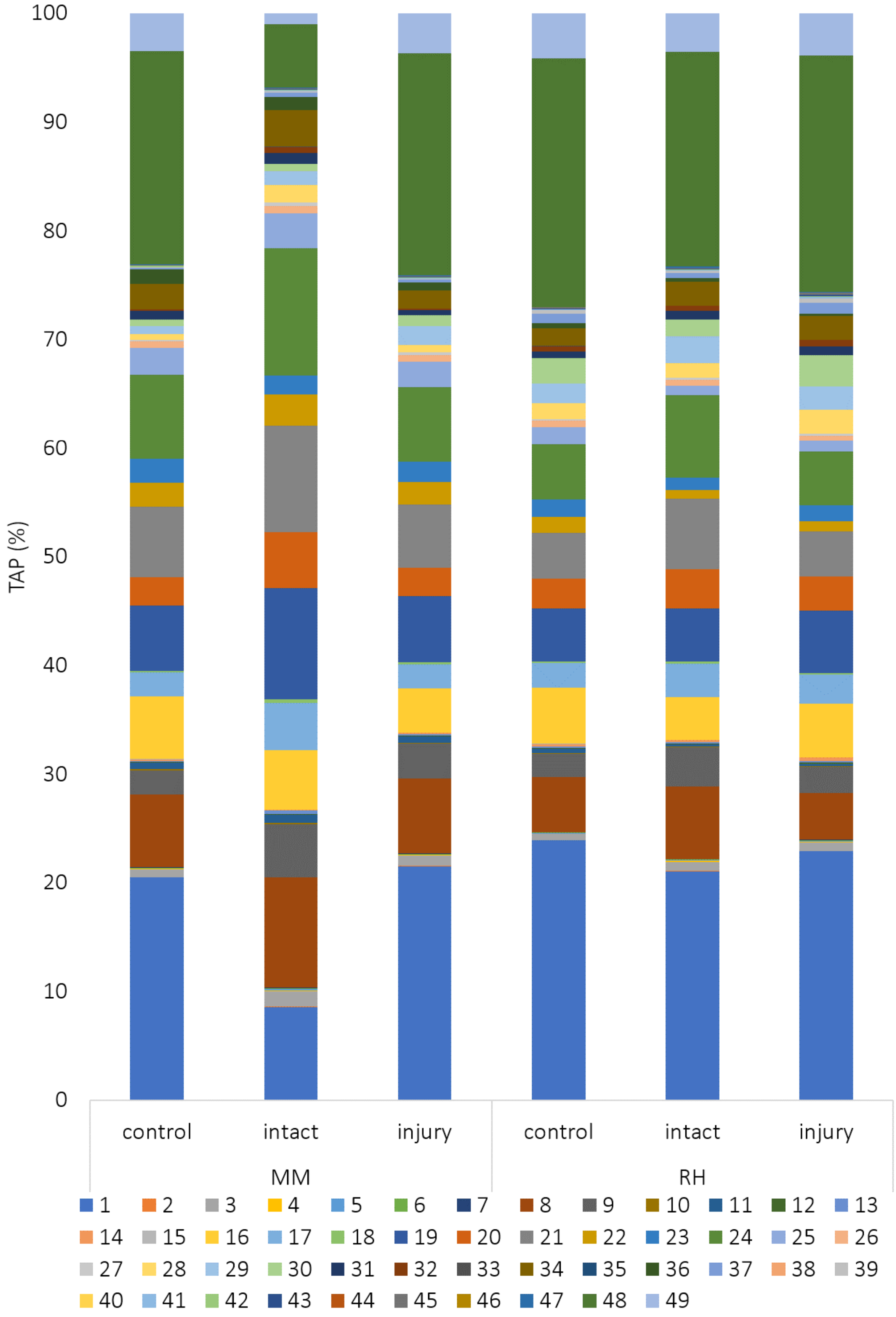

Figure S2: Proportion of individual phenolic compounds in total phenolic content (TAP) in the peach peels of different tissues, depending on the damage by a brown marmorated stink bug – control (tissue

from undamaged peaches), intact (undamaged tissue from damaged peaches) and injured (tissue of damaged peaches, from the injury zone in the surface depression). Two peach cultivars are presented – 'Maria Marta' (MM) and 'Redhaven' (RH). 1: neochlorogenic acid, 2: caffeic acid hexoside 1, 3: caffeic acid hexoside 2, 4: caffeic acid hexoside 3, 5: *p*-coumaric acid hexoside 1, 6: 3-*p*-coumaroylquinic acid, 7: *p*-coumaric acid hexoside 2, 8: chlorogenic acid, 9: 5-caffeoylquinic acid 2, 10: 3-feruloylquinic acid, 11: 4-caffeoylquinic acid, 12: 4-*p*-coumaroylquinic acid, 13: 5-*p*-coumaroylquinic acid 1, 14: 5-*p*-coumaroylquinic acid 2, 15: dicaffeoylquinic acid 1, 16: procyanidin dimer 1, 17: procyanidin dimer 2, 18: procyanidin dimer 3, 19: procyanidin dimer 4, 20: procyanidin trimer 1, 21: procyanidin trimer 2, 22: procyanidin timer 3, 23: procyanidin trimer 5, 24: catechin, 25: procyanidin tetramer, 26: epicatechin, 27: roseoside, 28: quercetin-3-rutinoside, 29: quercetin-3-galactoside, 30: quercetin-3-glucoside, 31: kaempferol-3-rutinoside, 32: quercetin-3-arabinofuranoside, 33: quercetin-3-xyloside, 34: isorhamnetin-3-rutinoside, 35: quercetin-3-glucuronide, 36: kaempferol hexoside 1, 37: kaempferol hexoside 2, 38: quercetin-3-arabinopyranoside, 39: isorhamnetin hexoside 1, 40: isorhamnetin hexoside 2, 41: quercetin-3-rhamnoside, 42: kaempferol acetylhexoside, 43: isorhamnetin acetylhexoside, 44: quercetin rhamnosyl hexoside 1, 45: quercetin rhamnosyl hexoside 2, 46: naringenin hexoside 1, 47: phloridzin, 48: cyanidin-3-glucoside, 49: cyanidin-3-rutinoside.
